# Pharmacodynamics of efavirenz 400 mg in treatment-naïve Chinese HIV-infected patients in a prospective cohort study

**DOI:** 10.1186/s12879-021-05802-8

**Published:** 2021-01-23

**Authors:** Ling Xu, Wenxiu Peng, Xiaojing Song, Yanling Li, Yang Han, Ting Zhu, Qiang Fu, Xiaoli Du, Wei Cao, Taisheng Li

**Affiliations:** 1 Department of Infectious Diseases, Peking Union Medical College Hospital, Chinese Academy of Medical Sciences, No.1 Shuaifuyuan, Wangfujing Street, Beijing, 100730 China; 2 Department of Pharmacy and Pharmacology, Peking Union Medical College Hospital, Chinese Academy of Medical Sciences, Beijing, China; 3 Clinical Immunology Center, Chinese Academy of Medical Sciences, Beijing, China; 4 Tsinghua University Medical College, Beijing, China

**Keywords:** EFV 400 mg, HIV RNA load, Plasma EFV concentration, HAMD, PSQI

## Abstract

**Background:**

The plasma concentration of patients treated with efavirenz (EFV) 600 mg was found to exceed the upper limit of the proposed therapeutic window in most Chinese HIV-infected individuals; thus, dosage reduction of EFV to 400 mg daily warranted consideration. This study aimed to assess the pharmacodynamics of EFV 400 mg for HIV-1-infected patients in China.

**Method:**

Twenty cART-naïve individuals were enrolled in this study. EFV 400 mg combined with tenofovir (TDF) and lamivudine (3TC) as an initial antiretroviral regimen was administered for 48 weeks. EFV concentration and T cell subsets as well as HIV RNA load were evaluated at baseline and at 4, 12, 24, and 48 weeks. Moreover, neuropsychiatric adverse effects were also assessed by the Hamilton depression (HAMD) scale and Pittsburgh sleep quality index (PSQI).

**Results:**

Eighteen males and two females whose median age was 26 (interquartile range [IQR]: 23–32) years completed 48 weeks of follow-up. The median EFV concentrations were 1.88 (IQR: 1.54–2.42), 1.74 (IQR: 1.36–1.93), 1.93 (IQR: 1.66–2.22), and 1.85 (IQR: 1.54–2.14) mg/L at weeks 4, 12, 24, and 48, respectively. The viral load was 4.59 (IQR: 4.10–5.19) log_10_ copies/mL at baseline, and it decreased by 4.6 (IQR: 3.98–5.18) log_10_ copies/mL from baseline to week 48. Three of 20 (15%), 10 of 20 (50.0%), 17 of 20 (85%), and 18 of 19 (95%) participants had a plasma viral load less than 50 copies/mL at weeks 4, 12, 24, and 48, respectively. The median CD4 cell count was 330 (IQR: 237–410) cells/μL at baseline, and it increased to 473 (IQR: 344–574) cells/μL at 48 weeks. The HAMD score was 5 (IQR: 3–9.8) and 3 (IQR: 2.25–4) at baseline and 48 weeks, respectively. The PSQI score was 4 (IQR: 2–5.8) and 3 (IQR: 2–4) at baseline and 48 weeks, respectively. Dizziness was the most common event, occurring in 70% of patients within the first 2 weeks of treatment.

**Conclusion:**

Patients prescribed with EFV 400 mg-containing agents demonstrated favourable virological and immunological responses. And the plasma EFV concentration was within the recommended therapeutic range, with fewer adverse reactions than with EFV 600 mg. EFV 400 mg was effective and safe in Chinese HIV-infected patients.

**Trial registration:**

NCT04596488; Registered 21 October, 2020; Retrospectively registered.

## Background

It was reported that 38 million people were living with HIV-1 and that 25.4 million patients were accessing combined antiretroviral therapy (cART) worldwide in 2019 [1]. Integrase strand transfer inhibitor (INSTI)-based regimens have been recommended as the first-line treatment due to good tolerance and few adverse events in most developed countries in recent years [2, 3]. Notably, nonnucleoside reverse transcriptase inhibitors (NNRTIs), such as efavirenz (EFV), play a key role in suppressing HIV RNA replication [4], and EFV combined with two nucleoside reverse transcriptase inhibitors (NRTIs) is still considered the preferred regimen in many developing countries, including China [5], because of its accessibility and cost efficiency.

However, neuropsychiatric adverse events, such as dizziness and nightmares, occur frequently in patients prescribed EFV, which forced many patients to replace EFV with other agents [6–8]. Interestingly, plasma EFV concentration, which was recommended at the range of 1–4 mg/L, was positively correlated with the occurrence of central nervous system (CNS) toxicity [9]. Patients with plasma EFV concentrations > 4 mg/L experienced three times more frequent CNS adverse events than those with concentrations in the recommended range [10]. Our previous multicentre study showed that the median plasma EFV concentration increased gradually over 48 weeks and that 43.8% of patients had an EFV concentration > 4 mg/L in China [11]. This result meant that HIV-infected Chinese adults were likely to suffer from neuropsychiatric symptoms. Therefore, it was necessary to choose a suitable dosage of EFV for Chinese patients to improve their treatment safety.

Some literature has revealed that EFV 400 mg is safe and effective in HIV-infected patients [12, 13]. However, many factors, such as genetic polymorphisms [14] and body weight [11], could significantly affect EFV metabolism, which is associated with the plasma EFV concentration. The association between plasma EFV concentrations with a 400 mg dose and virological and immunological responses in HIV-1-infected patients from China has rarely been reported by longitudinal follow-up studies. Moreover, researchers have usually characterized the prevalence of psychiatric events only by asking patients to self-report mood swings and insomnia [15] instead of by using scales, which are beneficial to identify depression and sleep disturbance more precisely than self-reporting. In this prospective study, we aimed to assess the pharmacodynamics of EFV 400 mg in 20 HIV-1-infected individuals commenced on EFV 400 mg combined with tenofovir disoproxil fumarate (TDF) and lamivudine (3TC) as an initial regimen. We observed the dynamics of plasma EFV 400 mg concentration and investigated the prevalence of CNS adverse events among these patients by the validated HAMD and PSQI scales over a 48-week period.

## Methods

### Subjects

We performed a prospective trial from June 2017 to December 2018 at the clinics of the Department of Infectious Disease in Peking Union Medical College Hospital (PUMCH). Eligibility criteria for adult participants included (1) HIV treatment-naïve, (2) age of 18 years or older, (3) willingness to complete the HAMD and PSQI scales and follow-up regularly, and (5) not participating in other studies. The exclusion criteria were as follows: (1) acute HIV-1-infected patients; (2) an AIDS-defining illness within 2 weeks of entry; (3) transaminase and alkaline phosphatase levels beyond three times the upper limit of the normal range, bilirubin level more than 2.5 times the upper limit of the normal range, and serum creatinine level in excess of 1.5 times the upper limit of the normal range; and (4) pregnancy or breast feeding.

### Procedures

Participants were assessed at baseline and weeks 4, 12, 24 and 48 for clinical adverse events, physical examination findings and biochemical analyses. Moreover, they were asked to complete the HAMD and PSQI scales at each visit.

### T cell subsets and HIV-1 RNA determination

CD4+ T lymphocytes and CD8+ T lymphocytes were determined by flow cytometry (FACS Canto, BD Biosciences, NJ, USA) using commercially available monoclonal antibodies, and plasma HIV-1 RNA load was measured using a COBAS Ampliprep/TaqMan 48 Real-time RT-PCR Test (Roche, CA, USA) according to the manufacturer’s instructions. The detection range was from 20 to 1,000,000 copies/mL. Viral suppression was defined as a plasma HIV-1 viral load below 50 copies/mL. Those who were not capable of achieving a plasma HIV-1 viral load < 200 copies/mL after 48 weeks of treatment were considered to experience virological failure.

### Plasma EFV concentration analyses

Blood samples were collected in ethylenediaminete-traacetic acid (EDTA) tubes at weeks 4, 12, 24 and 48 of EFV 400 mg treatment in the morning. After centrifugation, plasma samples were transferred to and stored at − 80 °C until analysis. EFV concentrations were assayed in plasma samples at the Department of Pharmacy and Pharmacology at PUMCH using a validated high-performance liquid chromatography method with ultraviolet (UV) detection, as described in a previous study [8].

### Assessment of depression and sleep quality

Trained clinicians evaluated depression by asking patients whether they had experienced the items that are included in the HAMD scale in the prior week. Patients with scores < 8 on the HAMD scale were considered to be normal, patients with scores of 8 to 20 tended to be depressed, patients with scores of 20 to 35 were thought to be depressed, and patients with scores > 35 were severely depressed. Participants also self-administered the PSQI to evaluate sleep disturbance during the prior month. A score > 5 on the PSQI was defined as sleep disturbance in this study.

### Ethics statement

The Institutional Review Board of PUMCH approved this study, and each participant provided written informed consent.

### Statistical analysis

Analyses were performed using SPSS 23.0 (IBM Corp, Armonk, NY, United States), and statistical significance was defined as a *p*-value < 0.05. Descriptive statistics are presented as the mean with standard deviation (SD) or median (M) with interquartile range (IQR). Student’s t-test was used for parametric data, and the Mann-Whitney U test was conducted for comparison of noncategorical variables. Categorical variables were analysed by the Chi-squared test or Fisher’s exact test. Associations between continuous variables were tested using a nonparametric Spearman rank correlation test.

## Results

### Characteristics of the study population

**Fig. 1:**
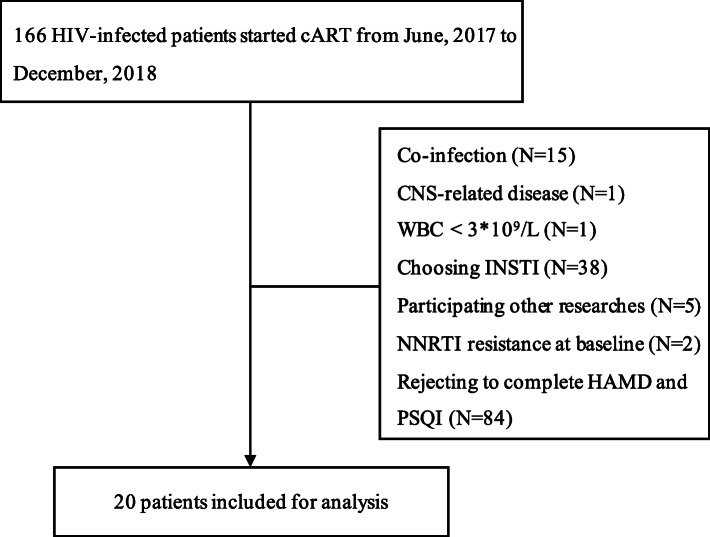
Flow of patients through the screening process

From a total of 166 cART-naïve patients seeking HIV care in PUMCH, 20 were eligible for this study. Reasons for exclusion are detailed in Fig. 1 and no significant difference was found in terms of CD4 cell count, CD8 cell count and HIV RNA level between the subjects included and excluded. The baseline characteristics of the enrolled patients were summarized in Table 1. The median age was 26 (IQR: 23–32) years, and the median body weight was 57 (IQR: 54–60) kg. Eighteen (90.0%) were male, and they were infected by homosexual contact.

**Table 1:** Characteristics of enrolled patients

Variables	*N* = 20
Age (years)	26 (23–32)
Men	18 (90.0%)
Transmission	
Homosexual contact	18 (90.0%)
Heterosexual contact	2 (10.0%)
Weight (kg)	57 (54–60)
Plasma HIV RNA (lg copies/mL)	4.59 (4.10–5.19)
HIV RNA (copies/mL)	
< 10,000	4 (20.0%)
10,000–100,000	10 (50.0%)
> 100,000	6 (30.0%)
CD4^+^ T cell count (cells/μL)	330 (237–411)
< 200	3 (15.0%)
200–350	10 (50.0%)
> 350	7 (35.0%)
CD8^+^ T cell count (cells/μL)	803 (678–1167)
cART regiments	
3TC + TDF + EFV	19 (95.0%)
3TC + ABC + EFV	1 (5.0%)

### Plasma EFV concentrations

**Fig. 2:**
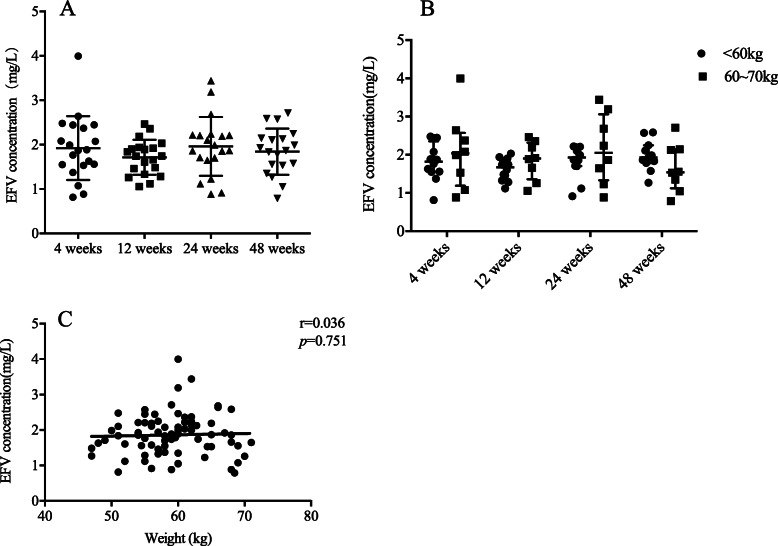
Dynamic changes to the plasma EFV concentration during cART treatment. The plasma EFV concentration in 79 samples **a** and patients stratified by body weight **b** and the correlation between weight and EFV concentration **c** were shown

Plasma sample collection time intervals were in the range of 8–20 h after the last dose of EFV in this study. Overall, 79 samples from 20 individuals were included for analysis. The median EFV concentrations at weeks 4, 12, 24, and 48 were 1.88 (IQR: 1.54–2.42), 1.74 (IQR: 1.36–1.93), 1.93 (IQR: 1.66–2.22), and 1.85 (IQR: 1.54–2.14) mg/L, respectively. No significant difference was found in EFV concentration at any time-point (*p* > 0.05). Moreover, the EFV concentrations of 93.67% (74/79) of the samples were within the proposed therapeutic window of 1.0–4.0 mg/L. Only 6.33% (5/79) of patients had plasma EFV concentrations < 1.0 mg/L, at 0.884, 0.814, 0.916, 0.885, and 0.79 mg/L. None of the patients had EFV concentrations > 4.0 mg/L (Fig. 2a).

When stratified by baseline body weight, patients with body weight < 60 kg had EFV concentrations similar to that of patients with body weight > 60 kg at each study time-point (Fig. 2b). Additionally, further correlation analysis showed that there was no significant association between weight and EFV concentration at every study time-point (Fig. 2c).

### Efficacy

**Fig. 3:**
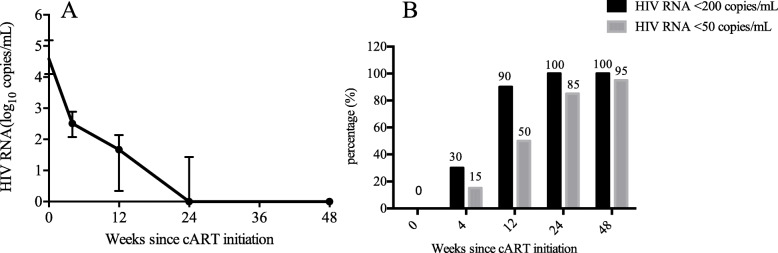
Virological responses in HIV-infected individuals commenced on EFV 400 mg. The decrease of HIV RNA load **a** and proportion of patients who achieved virological suppression at different time-points **b** were recorded

**Fig. 4:**
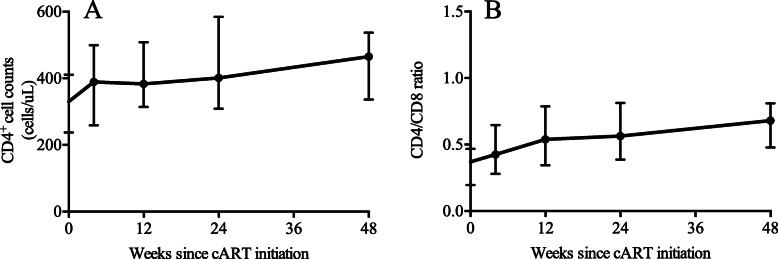
Immunological responses in HIV-infected patients. The graphs demonstrated the increase of CD4 cell counts **a** and CD4/D8 ratio during follow-up period **b**

Of the enrolled patients, the median HIV RNA load was 4.59 (IQR: 4.10–5.19) log_10_ copies/mL at baseline. As expected, the largest decrease in HIV RNA occurred during the first 24 weeks, and the median decrease from baseline was 4.24 (IQR: 3.98–5.18) log_10_ copies/mL. At 48 weeks, the median decrease from baseline was 4.6 (IQR: 3.98–5.18) log_10_ copies/mL (Fig. 3a). The proportions of participants with a viral load below 50 copies/mL were 15% (3/20), 50% (10/20), 85% (17/20), and 95% (18/19) at weeks 4, 12, 24, and 48, respectively (Fig. 3b). Furthermore, the median CD4 cell count was 330 (IQR: 237–411) cells/μL at baseline and increased by 133 (IQR: 49–223) cells/μL at week 48 (Fig. 4a). The CD4/CD8 ratio also experienced a rising trend, from 0.37 (IQR: 0.2–0.47) at baseline to 0.68 (IQR: 0.48–0.81) at week 48 (Fig. 4b). Specifically, three patients achieved normalization of the CD4/CD8 ratio (CD4/CD8 > 1) at the end of follow-up.

### Adverse events

**Fig. 5:**
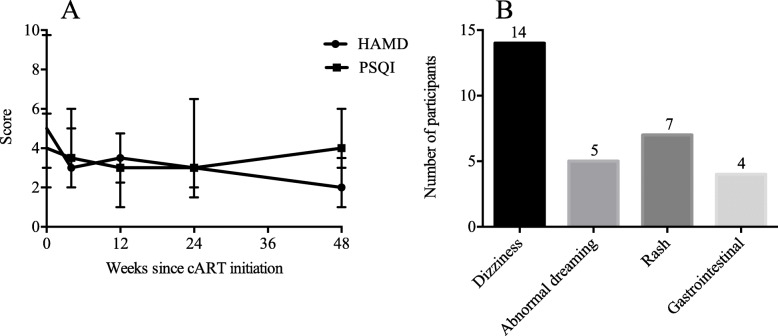
The changes of safety parameters. The trajectory of score on HAMD and PSQI **a** and the occurrence of adverse events **b** were displayed in details

Overall, the median HAMD scores were 5 (IQR: 3–9.8), 3 (IQR: 2–6), 3.5 (IQR: 1.0–4.8), 3 (IQR: 2–6.5), and 2 (IQR: 1–3.5) at baseline, week 4, week 12, week 24, and week 48, respectively. Six patients were likely to be depressed at baseline, and the condition did not progress during the follow-up. The median PSQI scores were 4 (IQR: 2–5.8), 3.5 (IQR: 2–5), 3 (IQR: 2.3–4.8), 3 (IQR: 1.5–6.5), and 4 (IQR: 3–6) at baseline, week 4, week 12, week 24, and week 48, respectively. None of them experienced poor sleep throughout the duration of follow-up (Fig. 5a).

Dizziness was the most frequent symptom, and one patient stopped taking EFV because of dizziness at week 36. Rash, abnormal dreaming, and gastrointestinal injury occurred in 7, 5, and 4 patients, respectively, during the 48 weeks of follow-up (Fig. 5b).

**Fig. 6:**
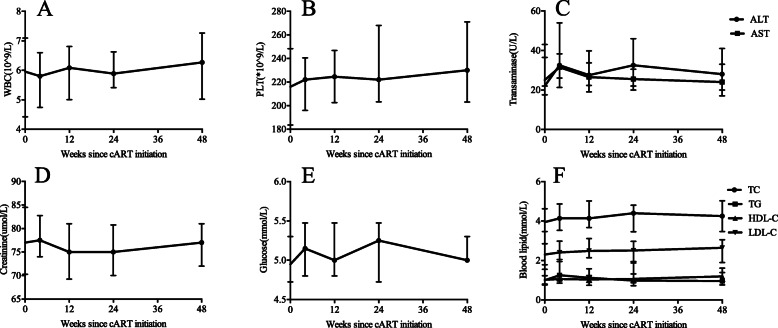
The dynamics of biochemical or haematological parameters. No significant change was observed in terms of WBC counts **a**, PLT counts **b**, transaminase **c**, Creatinine **d**, Glucose **e** and blood lipid levels **f** during follow-up period

White blood cell, lymphocyte, neutrophil and platelet counts were within the normal range from baseline to week 48 (Fig. 6). Alanine aminotransferase was 88 U/L in one patient, and total bilirubin was 39.6 mmol/L in another patient at baseline. However, both recovered at week 48.

## Discussion

Guidelines in many developed countries have considered INSTI-based regimens as first-line therapies for HIV-1-infected individuals [2, 3]. However, the utility of INSTIs is restricted in developing countries because of the relatively high expenditure. At present, EFV-containing regimens are commonly used in China even though some patients are disturbed by CNS adverse effects of EFV. The efficiency and safety of EFV dose reduction in HIV-infected patients have been proven in some studies [12, 13], but the dynamics of EFV concentrations with a 400 mg dose have rarely been reported in longitudinal studies, which were associated with the occurrence of EFV adverse events. In this prospective, single-arm study, we first demonstrated that EFV 400 mg combined with two NRTIs as initial antiretroviral therapy maintained the recommended EFV plasma concentration over 48 weeks in Chinese patients, many of whom were infected with HIV by homosexual contact. Moreover, patients treated with EFV dose reduction experienced fewer neuropsychiatric adverse events than those with EFV 600 mg therapy, as measured by the credible HAMD and PSQI scales. These results provide support for the widespread use of EFV 400 mg in China, which could facilitate the optimization of current treatment for HIV-1-infected patients.

Our previous study indicated that nearly half of Chinese patients who were treated with EFV 600 mg-containing cART had EFV concentrations above the upper limit of the proposed therapeutic window, especially those with body weights less than 60 kg [11]. Nyakutira et al. also showed that EFV plasma concentrations were above 4 mg/L in 50% of patients receiving EFV 600 mg in Zimbabwe [16]. Based upon these findings, a dose reduction of EFV to 400 mg seemed reasonable. In our study, we found that the EFV concentration of 93.67% of the samples was in the recommended range. Additionally, the concentration at 48 weeks was not higher than that at 4 weeks, 12 weeks, and 24 weeks, suggesting that the reduced EFV dose would not lead to drug accumulation. This characteristic was different from the results with EFV 600 mg, which showed that the concentration at 4 weeks was lower than that at 24 weeks and 48 weeks. We suspected that the half-life of EFV was up to 40–80 h [17]; thus, dose reduction could be beneficial for less drug accumulation. Additionally, the serum concentration with the reduced dose was not beyond 4 mg/L. In patients who took EFV 600 mg, there were 45.28% patients whose serum concentration was more than 4 mg/L at 48 weeks. Taken together, these results indicated the safety of the dose reduction.

However, the serum EFV concentration of three patients was less than 1 mg/L during follow-up, and they all achieved full virological suppression in the end. Dickinson et al. proposed that EFV concentration cut-offs between 0.47 and 0.76 mg/L provided acceptable sensitivity and specificity criteria for virological failure [18]. The lowest concentration in our study was 0.79 mg/L, higher than the proposed cut-off range, suggesting that the serum EFV concentration in our study was virologically effective.

Previous literature also showed that weight > 60 kg was associated with lower EFV concentrations in HIV-infected patients [11, 19]. In our study, the weight of patients at baseline ranged from 47 to 67 kg. We found that individuals whose weight was more than 60 kg had EFV concentrations comparable to those of patients whose weight was less than 60 kg. Weight was not linked to the concentrations of the reduced dose, suggesting the EFV 400 mg dose may be appropriate across a wide weight range. However, further studies are needed to confirm this result because we did not include patients with higher weights, such as more than 70 kg.

At week 48, 96.9% of HIV-infected patients treated with EFV 600 mg-containing cART had virological suppression (< 40 copies/mL) [20]. The ENCORE1 study revealed that 94.1% of cART-naïve HIV-infected individuals achieved a viral load below 200 copies/mL in the EFV 400 mg group [13]. Encouragingly, in our study, 95% of patients had a viral load less than 50 copies/mL, and all patients had a viral load below 200 copies/mL at 48 weeks. These consequences were similar to the above results, indicating the potency of the reduced dose. Notably, we found that CD4 cell counts increased by 133 (IQR: 49–223) cells/μL at week 48, which were higher than the counts observed in the ENCORE1 study. The difference may be the consequence of higher baseline CD4 cell counts in our study, which was positively associated with the recovery of CD4 cell counts after cART initiation [21, 22]. These results suggested the efficacy of EFV 400 mg and provided support for the widespread use of a reduced dose in HIV-infected Chinese patients, especially in those infected with HIV by homosexual contact.

The common EFV-related CNS adverse events were dizziness, abnormal dreams, insomnia, and depression [23, 24]. It was reported that 50% of patients who were prescribed EFV 600 mg experienced these events, which usually occurred in the first month of treatment and continually existed [25, 26]. We found that 70% (14/20) and 25% (5/20) of patients suffered from dizziness and abnormal dreams in the preliminary two weeks in our study, which was in agreement with previous studies [25, 26]. After that, patients self-reported that discomfort occurred less often, except for one patient who had drug discontinuation. We assumed that the cessation of symptoms may be associated with improved tolerance and less drug accumulation.

The PSQI includes a 19-item questionnaire that evaluates sleep quality and efficiency during the prior month [27]. The HAMD scale is one of the most widely applied clinical measures of depression in psychiatric studies and has strong psychometric reliability and validity [28, 29]. Thus, we assessed sleep quality and depression by the PSQI and HAMD scales in this study, finding that the quality of sleep in participants was satisfactory and that no participants developed depression. This finding was not consistent with previous literature documenting symptoms of depression existing in 32.9% of HIV-infected adults on treatment in China [30]. This discrepancy may be due to the following reasons. First, dose reduction contributed to reducing CNS toxicity and improving patients’ long-term tolerance. The second reason was associated with the availability of free antiretroviral regimens and considerate medical care in our study, whereas an earlier study included patients with limited medical resources.

Our study has several limitations that warrant mention. First, this is a pilot study with a limited sample size and thus limited power. Therefore, a randomized, multicentre study should be performed to further investigate the efficacy and safety of EFV 400 mg in HIV-infected individuals from China. Second, we were unable to measure the frequency of the G516T polymorphism [14, 31–34], which has been linked with elevated plasma EFV concentrations, among our study participants. Future studies should be conducted to evaluate the distribution of the G516T polymorphism and its effect on serum EFV concentrations in HIV-infected adults treated with EFV 400 mg from China.

## Conclusion

Our study preliminarily demonstrated that EFV 400 mg-containing regimens were successful in suppressing HIV RNA replication and rebuilding immunological function in HIV-infected Chinese patients. Importantly, the plasma EFV concentration remained within the normal range, and CNS toxicity was reduced. These results suggested the efficacy and safety of EFV 400 mg in HIV-infected individuals from China, providing more appropriate treatment for these patients.

## Data Availability

Datasets used in this analysis are available from the corresponding author on reasonable request.

## Abbreviations

cART: Combined antiretroviral therapy
HAMD: Hamilton depression scale
PSQI: Pittsburgh sleep quality index
PUMCH: Peking Union Medical College Hospital
INSTI: integrase strand transfer inhibitor
