# Correction to: Pharmacodynamics of efavirenz 400 mg in treatment-naïve Chinese HIV-infected patients in a prospective cohort study

**DOI:** 10.1186/s12879-021-05880-8

**Published:** 2021-02-17

**Authors:** Ling Xu, Wenxiu Peng, Xiaojing Song, Yanling Li, Yang Han, Ting Zhu, Qiang Fu, Xiaoli Du, Wei Cao, Taisheng Li

**Affiliations:** 1 Department of Infectious Diseases, Peking Union Medical College Hospital, Peking Union Medical College and Chinese Academy of Medical Sciences, Beijing, China; 2 Department of Pharmacy and Pharmacology, Peking Union Medical College Hospital, Chinese Academy of Medical Sciences, Beijing, China; 3 Clinical Immunology Center, Chinese Academy of Medical Sciences, Beijing, China; 4 Tsinghua University Medical College, Beijing, China

**Correction to: BMC Infect Dis 21, 112 (2021)**

**https://doi.org/10.1186/s12879-021-05802-8**

After publication of the original article [1], the authors identified an error in the first affiliation.

The incorrect affiliation is:

Department of Infectious Diseases, Peking Union Medical College Hospital, Chinese Academy of Medical Sciences, No.1 Shuaifuyuan, Wangfujing Street, Beijing 100730, China

The correct affiliation is:

Department of Infectious Diseases, Peking Union Medical College Hospital, Peking Union Medical College and Chinese Academy of Medical Sciences, Beijing, China

The original article has been corrected.
